# Cases from Practice

**Published:** 1872-12-01

**Authors:** Wm. E. Quine


### Original Communications.

### CASES FROM PRACTICE.

BY WM. E. QUINE, M. D.

I.—*Phlebitis caused by a fall: Recovery:* In December, 1871, Mrs. B———, in the eighth month of her fifth pregnancy fell down a short flight of stairs, striking heavily on her sacrum. She experienced intense pain at the time, in the injured part, which was much aggravated by attempts at walking; but in the course of two or three hours this had almost entirely disappeared. About four hours after the accident, while resting quietly in a large chair, she was attacked suddenly with excruciating pains in the right thigh, at a point corresponding with the saphenous opening. A few minutes later, pain of a like character and intensity was experienced in the flexure of the knee—and then on the inner surface of the foot. So violent was the agitation of the patient, that the husband in alarm sent for the nearest physician; who, regarding the case as one of neuralgia, directed the application to the whole anterior and inner surface of the thigh of a powerful irritant. I was summoned to the bedside the following morning, and found the patient in a very alarming condition.

Her manner was hurried and agitated; her countenance expressive of anxiety and alarm; pulse about 180; respiration 38; and temperature 102½. The affected thigh was, from the irritant that had been applied, excessively tender, and the skin highly inflamed. Directly over the saphenous opening there was a nodular and exquisitely sensitive swelling; and the internal saphenous vein, throughout its whole course, felt hard, and pressure on it elicited complaints of suffering. The patient, though a woman of great fortitude, was filled with apprehensions of death; and she wept almost constantly at the idea of early parting with her young family, and at the fate of her unborn child. In accordance with my conception of the indications, I ordered leeches to be applied along the course of the inflamed vein; but so great was the patient's repugnance for them, that I was unable to overcome it, by assurances of immediate relief and permanent advantage.

Warm fomentations were then prescribed, sufficient morphine to procure rest, and a sedative mixture, in which tinct. veratri viridis was the most important ingredient. The treatment was faithfully continued through thirty-six hours; but then, on the advice of Dr. T. D. Fitch, whom I had called in consultation, the sedative was discontinued. The pain in the thigh, of which the patient had complained, gradually subsided, but the foot and leg became swollen and more painful than ever. The fomentations were then replaced by linseed poultices, and the latter were continued for a week; at the end of which, a point of fluctuation having been discovered, over the internal malleolus, a free incision was made, and a large quantity of pus evacuated. From this time the condition of the patient steadily improved, though it was six or seven weeks before she entirely recovered.

II.—*Rupture of the Utero-Placental Adhesions by a Fall: Septicæmia and Death:* In October of this year I was summoned to bedside of a young primipara, in the eighth month of pregnancy. She had enjoyed uninterrupted health up to the day preceding my visit, and had labored hard and continuously. At the time mentioned, while carrying a heavy burden down stairs, she slipped and fell, straining her back severely; and immediately after experienced sensations of nausea, and commenced to vomit. The vomiting continued at short intervals throughout the night, and was associated with dull, grinding, irregular pain in the hypogastrium. I found the patient in the morning much exhausted by the protracted emesis, and though her skin felt natural, her pulse was quick, small, soft, and 130 in the minute; she was writhing constantly, as if in severe pain, and the hand on the hypogastrium readily distinguished through the thinned abdominal wall, irregular uterine contraction; the bowels were constipated; and the patient, though she experienced a constant desire to urinate, had been unable to evacuate the bladder. The fœtal circulation, and the placental souffle were inaudible. On these grounds was based the diagnosis standing at the head of this history.

The patient was catheterized; her rectum cleared by an enema; opium, subnitrate of bismuth, and acetate of lead were administered in full doses, and at short intervals, and a sinapism was applied over the sacrum. In the evening, though the vomiting had ceased, the suffering of the patient seemed to have increased; the pain was more acute and continuous. I now noticed, for the first time, a reddish-purple discoloration of the face, and suffusion of the eyes, but attributed it to venous congestion consequent on voluntary expulsive efforts. The pulse had gained in frequency, and lost in force, since morning. A vaginal examination revealed the fact that dark, offensive, grumous blood was slowly issuing from the uterus. On the following morning the discoloration of the face had reappeared, and was attended with slight swelling, and tenderness of the integument. So striking was its appearance that I was led to examine other parts of the body, and actually found the whole surface covered with a purplish rash, interspersed with blackish points. The body at this time was bathed in perspiration; the pulse 154; temperature 104; respiration 40. There was no swelling or soreness of the throat; no cough, or physical sign of pulmonary disease; no albumen in the urine, or deficiency in quantity of the latter; the patient was ignorant of exposure to any infectious disease, and had had scarlatina, measles, and varioloid, in her childhood. Though it was now obvious that the patient would die, I prescribed quinine gr. ij.: strychnine gr. 1-32: nitric acid gtts. iv.: every three hours; and a tablespoonful of sherry wine every hour. In the evening, I met in consultation Drs. T. A. Clark, and D. C. Stillians, both of whom concurred in the diagnosis, and ratified the treatment. During the consultation the question of delivering the patient was introduced, but the proceeding was not deemed advisable. It may seem surprising, in view of the supposed death of the fœtus, that artificial delivery was not attempted at an early period in the history of the disorder. But at no time did I consider interference of this kind proper or justifiable; and this opinion was founded on the prevalence in the neighborhood of a malignant form of puerperal fever. The rash which appeared first on the face, then on the trunk and extremities, is perhaps the most interesting and curious feature of the case. And its interest is enhanced by the fact that recently a case was reported to the Chicago Medical Society, as malignant scarlatina, following natural delivery, in which the eruption, judging from the description given, was a counterpart of this. I do not regard the appearance of a scarlet rash during the course of puerperal fever, or septicæmia from other conditions, as positive evidence of scarlatinous infection; for I have several times witnessed it in cases in which there had been no possibility of recent exposure; and in patients who had previously had the disease, well marked. Moreover, the entire absence of the characteristic local diseases of scarlatina confirms this view.

